# Genome-wide association study uncovers key genomic regions governing agro-morphological and quality traits in Indian mustard [***Brassica juncea*** (L.) Czern. and Coss.]

**DOI:** 10.1371/journal.pone.0322120

**Published:** 2025-04-24

**Authors:** Manoj Kumar Patel, Navinder Saini, Yashpal Taak, Sneha Adhikari, Rajat Chaudhary, Priya Pardeshi, Sudhakar Reddy Basu, Masochon Zimik, Sangita Yadav, K. K. Vinod, Sujata Vasudev, Devendra Kumar Yadava

**Affiliations:** 1 Division of Genetics, ICAR-Indian Agricultural Research Institute, New Delhi, India; 2 Division of Seed Science and Technology, ICAR-Indian Agricultural Research Institute, New Delhi, India; 3 Indian Council of Agricultural Research, New Delhi, India; ICAR-Indian Institute of Pulses Research, INDIA

## Abstract

In Indian mustard, improving agro-morphological and quality traits through conventional methods are both cumbersome and resource-intensive. Marker-aided breeding presents a promising solution to these challenges. Hence, the present research aimed to identify genomic regions governing agro-morphological and quality traits using genome-wide association studies (GWAS). The GWAS panel comprised 142 diverse genotypes of Indian mustard were evaluated for 20 different agro-morphological and quality traits, revealing significant difference among genotypes. Subsequently, the GWAS panel genotyped using the *Brassica* 90K SNP array (Illumina). Structure and diversity analysis grouped the GWAS panel into 3 sub-populations or groups, and LD decay of 1.05 Mb was confirmed through genotypic analysis. GWAS using the BLINK model revealed a total of 49 marker-trait associations (MTAs), in which 28 and 21 MTAs were observed during *rabi* 2020–21 and *rabi* 2021–22 for various agro-morphological and quality traits, respectively. Amongst them, twelve MTAs demonstrated stable associations with the studied traits, including days to 50% flowering (DF), days to 100% flower termination (DFT), days to maturity (DM), plant height (PH), main shoot length (MSL), siliqua length (SL), seeds per siliqua (SPS), oil content (OC), and glucosinolates content (Glu) in both years. Moreover, *in silico* analysis of nearby regions of these stable SNPs revealed their association with 31 candidate genes known to be involved in various molecular, physiological, and biochemical pathways relevant to the studied traits. These genes can be further characterized and deciphered for more precise utilization in breeding programs in the future.

## Introduction

Indian mustard [*Brassica juncea* (L.) Czern. & Coss.; AABB; 2n=36], is an amphidiploid species of Brassicas belonging to the family Cruciferae. It is predominantly a self-pollinating crop; however, based on bee activities, up to 18% cross-pollination has been reported under natural field conditions [1]. Indian mustard is a predominant species among the rapeseed-mustard group of crops in India and accounts for more than 90% of its total acreage [2]. It is mainly used for edible oil, especially in India, China, and European countries. In addition, it is also utilized as condiments, salad, leafy vegetables, green manure, fodder, and biofuels [3,4]. Globally, India holds the third position in acreage as well as production after Canada and China. During 2022–23, the world’s rapeseed-mustard seed production was about 87.06 million tones (mt) in 41.08 million hectares (mha) area with productivity of 2.08 t/ha [5]; whereas, in India, rapeseed-mustard production was 11.70 mt from an area of 9.2 mha with an average productivity of 1.27 t/ha [5]. Therefore, there is a huge scope (~0.81 t/ha) of improvement in seed productivity under Indian conditions.

In the context of Brassicas, the main challenge for present-day plant breeders is to develop high-yielding varieties to meet the demands of growing global populations. Therefore, breeding programmes of Indian mustard are mainly targeting improvement in seed productivity and oil quality. In Brassicas, seed yield is determined by various agro-morphological traits such as plant height, main shoot length, siliquae per plant, siliqua length, seeds per siliqua, etc. [6,7]; thus, these traits have significant role in improving the seed yield. On the other hand, oil or meal quality is determined by fatty acids, proteins, and glucosinolates constitution [4,8]. Amongst these, the fatty acid composition is a chief determinant of oil quality [9]. It has been reported that elevated amounts of saturated fats and erucic acid resulted in high cholesterol levels in humans [10]. Additionally, glucosinolates, another important determinant of oil quality, are considered as an anti-nutritional factor in animal feed, which exerts adverse effects on animals if present in a high proportion [11]. Therefore, in Brassicas, these traits are highly crucial for the improvement of oil quality. However, the desired modification of these traits using conventional means is very challenging and resource-demanding due to their complex inheritance and environmental influence. Therefore, the identification of genomic regions/quantitative trait loci (QTL) is of prime importance, as it will further aid in molecular breeding for efficient and accelerated crop improvement.

Advancements in molecular techniques have led to the evolution of markers from SSR to DArT and ultimately SNPs, which are now widely used in applications like QTL mapping, GWAS, and genomic selection [12,13]. Genotype-by-sequencing (GBS) is a popular SNP genotyping method, but high percentage of missing values, low sequencing coverage, unknown marker positions, and complex statistical analysis make its use limited [14,15]. These limitations are effectively addressed by array-based SNP genotyping, offering improved reliability and ease of analysis [16,17]. QTL mapping using biparental populations has limited precision due to restricted recombination, requiring additional efforts for fine mapping [3,18]. In contrast, Association Mapping (AM) utilizes natural populations and historical recombination, providing higher resolution and capturing a broader genetic variation. GWAS, a key AM approach, has become vital for identifying the genetic basis of complex traits in crops [19–21]. The physical extent of linkage disequilibrium (LD) around a gene significantly influences the efficiency of association mapping. This extent is shaped by various factors, such as the rate of outcrossing, selection pressure on specific genomic regions, recombination rates, chromosomal location, and population size and structure [22]. Therefore, accounting for these factors is essential to ensure reliable results in association mapping. With the increasing adoption of whole-genome sequencing (WGS), the power and resolution of GWAS have been greatly enhanced [23]. Consequently, association mapping using SNP markers has emerged as one of the most effective strategies for identifying genomic regions and candidate genes.

In crucifers, several candidate genes associated with various agro-morphological and quality traits have been reported. For instance, *WRKY* transcription factors and *BTB/POZ* domain family genes (*BOP* genes) regulate flowering time in *Arabidopsis thaliana* [24,25]. Additionally, *Hd3b*, a rice ortholog of the *EFL3* gene in *A. thaliana*, has been found to cause delayed flowering under long-day field conditions in rice [26]. Genes such as *IAA17* and *B-box zinc finger protein 24* are involved in plant growth and development [27,28]. In *B. napus*, Pal et al. [29] identified the candidate gene *GATA15* associated with seeds per siliqua. For quality traits, *WRI1* and oleosin genes play a crucial role in oil accumulation [30,31]. In *Brassica juncea*, the *FAE* gene regulates erucic acid content [32,33] while, *MYB28*, *MYB29*, *CYP79F1*, *GSL-ELONG* and *GSL-ALK* genes are associated with glucosinolates biosynthesis [34]. Moreover, in Brassicas, GWAS played a paramount role in identifying the important gene(s) or genomic regions for different agro-morphological and quality parameters including flowering traits [35], plant height [35,36], primary branches per plant [36], siliqua length [37], seeds per siliqua and thousand seed weight [38], seed yield components [3,29,39], oil content [4,40,41], erucic acid [42] and glucosinolates [4,43,44]. However, in *B. juncea*, only a few reports on GWAS for agro-morphological [3,35,39] and quality traits [4,45] are available and to date, no study on GWAS using array-based SNP genotyping has been reported*.*

Building on this background, the present study utilized a germplasm panel of 142 genotypes for GWAS to identify MTAs and candidate genes for key agro-morphological and quality traits. The panel was evaluated across two consecutive seasons (*rabi*, 2020–2021 and *rabi*, 2021–2022) and genotyped using the *Brassica* 90K SNP array (Illumina). The study identified important stable MTAs, along with associated candidate genes, which hold great potential for advancing molecular breeding efforts in Indian mustard.

## Materials and methods

### Germplasm panel for GWAS

The GWAS panel consisting of 142 diverse genotypes of Indian mustard contains Indigenous lines, developed varieties, exotic lines, advanced breeding material as well as introgressed lines, which provided from Panjab Agricultural University, Ludhiana collected under the ICAR - National Agricultural Science Fund (NASF) project (S1 Table).

### Design of experiments

Germplasm assembly of 142 Indian mustard genotypes was grown at research farm, ICAR-Indian Agricultural Research Institute, New Delhi during *rabi,* 2020–21 and *rabi*, 2021–22 in augmented block design with seven checks (DRMRIJ-31, PM-30, RLC-3, RH-749, NRCHB-101, PM-25 and PM-28) and four blocks. Check varieties included conventional as well as quality mustard varieties (low erucic acid/glucosinolates content, i.e., single zero and double zero type). In both seasons, each genotype had grown in two rows of 5m length with 45 cm × 15 cm spacing. The germplasm panel was evaluated for different agro-morphological and quality traits. To ensure better crop establishment, two irrigations were provided using the flood method. The first irrigation was applied at 35–40 days after sowing (DAS), and the second at 85–90 DAS. Fertilizers were applied at rates of 60 kg/ha N, 40 kg/ha P, and 40 kg/ha K. Phosphorus and potassium were incorporated into the soil prior to sowing, while nitrogen was top-dressed at various growth stages. All other agronomic practices, including thinning and weeding, were carried out following the recommended package of practices.

### Record of observations

Observations in the present investigation were recorded on 12 different agro-morphological traits, which include days to 50% flowering (DF), days to 100% flower termination (DFT), days to maturity (DM), plant height at maturity (PH; cm), main shoot length (MSL; cm), primary branches per plant (PBPP), secondary branches per plant (SBPP), siliquae per plant (SPP), siliqua length (SL; cm), seeds per siliqua (SPS), seed yield per plant (SYPP; g), biological yield per plant (BYPP; g) and 8 oil quality traits, i.e.*,* oil content (OC; %), glucosinolates (Glu; µmol/g), and fatty acids content (%) namely, palmitic acid (PA), oleic acid (OA), linoleic acid (LliA), linolenic acid (LlnA), eicosenoic acid (EcA) and erucic acid (ErA). Observations for DF, DFT, and DM were recorded from the whole plot; while that of remaining traits were documented on five randomly selected plants from each genotype. Five randomly selected plants of each genotype were harvested at the time of maturity and sun-dried until no residual moisture remained, then using weighing balance BYPP was recorded in grams. After the record of biological yield, plants were threshed manually and their seed yield was recorded as SYPP using a weighing balance. Furthermore, phenotyping of seed-related parameters such as OC, Glu, and fatty acid contents was carried out on freshly threshed seeds from each selected plant of a genotype. Oil content has been estimated by the non-destructive method using Near InfraRed spectroscopy (Perten, DA 7250). Glucosinolates content was estimated by the simple spectrophotometric method suggested by Mawlong et al. [46]. In fatty acid profiling, the preparation of methyl esters was carried out as per the standard protocol suggested by Vasudev et al. [8]; while fatty acid peaks were captured on Gas Chromatograph (Perkin Elmer Claurus 500) and the amount of fatty acid is calculated by triangulation method.

### Molecular analysis and SNP genotyping

Genomic DNA was extracted from tender leaves of 142 mustard genotypes using the CTAB (Cetyl Trimethyl Ammonium Bromide) method [47]. The DNA quality was analyzed using a 0.8% agarose gel with a known and standard DNA (uncut lambda DNA) and samples were quantified using Nanodrop (NanoDrop 2000, Thermo Scientific, USA). Approximately 5µg DNA from each genotype was used for SNP genotyping using Brassica 90K SNP array, Illumina iScan, Infinium assays (AgriGenome Labs Pvt. Ltd.). A total of 77970 SNPs were obtained. Subsequently, genotypic data was subjected to quality check and filtering, wherein 15219 high-quality, polymorphic SNPs with a minor allele frequency of more than 5% and missing data less than 10% were retained for Linkage disequilibrium (LD) and GWAS analysis (S2 Table). However, for structure, diversity, and analysis of molecular variance (AMOVA), markers that are closely located and are in LD (<1.05 MB in the present study) were removed and 620 widely distributed SNPs, at a distance of ~1.05 MB were used.

## Data analysis

### Phenotypic evaluation of GWAS panel

The season-wise estimation of the analysis of variance (ANOVA) and descriptive statistics of the phenotypic data were performed using the “augmented RCBD” R package (Version 4.2.2) [48]. Individual and combined best linear unbiased predictors (BLUPs) across the seasons (*rabi*, 2020–2021 and r*abi*, 2021–2022) were analyzed using software PB tools (version 1.4. 2014, *Biometrics and Breeding Informatics, PBGB Division, IRRI*) considering genotypes as random effects. Subsequently, Individual BLUP values were used as phenotypic input for GWAS analysis. Whereas, combined BLUPs were used for Principal Component Analysis (PCA) of phenotypic data using R packages FactoMineR and Factoextra [49,50].

### Structure, diversity, and AMOVA of GWAS panel

Identified 620 SNPs were subjected to structure analysis using structure software (v2.3.4) [51]. The allele admixture model was used for structure analysis, wherein the burn-in period and MCMC repeats were kept at 1,00,000. The number of iterations was kept three and the number of sub-populations (k) ranged from 1 to 10. An appropriate number of delta k (sub-populations) was determined using Evanno method [52] with the help of the online tool Structure Harvester (http://taylor0.biology.ucla.edu/structureHarvester). To estimate the variance within and among sub-population, analysis of molecular variance (AMOVA) was performed using R based package “Poppr” [53]. Thereafter, neighbor-joining tree was constructed using Tassel 5 (v5.2.75) [54] for studying molecular diversity using the same set of SNPs.

### LD decay analysis

Linkage disequilibrium (LD) values of filtered SNPs were estimated using the *r*^*2*^ method *via* software Tassel 5 (v5.2.75) [54]. Further, the LD decay plot was obtained by plotting *r*^*2*^ values against the physical distance between markers (in bp) using the R package as per [55] and a standard cutoff value of *r*^*2*^ = 0.1 was followed to estimate LD decay [56].

### GWAS analysis

GWAS analysis was done using R based package “Genome Association and Prediction Integrated Tool” (GAPIT) [57] using Bayesian-information and linkage-disequilibrium iteratively nested key-way model (BLINK) [58]. PCA using genotypic data is a good indicator of population structure in association mapping. Therefore, the first three principal components were generated and used as covariate in GWAS analysis using the GAPIT tool. The fitness of the GWAS model was determined by Q-Q plot plotted between observed and expected -log_10_ (*p*) values. The threshold for significant marker-trait associations (MTAs) was determined using Bonferroni corrected *p*-value (effective threshold of -log 10 (*p*) = 5.48). However, for quantitative traits, Bonferroni threshold can be too conservative due to the involvement of minor genes [59,60]. Therefore, a less stringent criterion (*p*-value <0.001) was adopted to identify stable SNPs.

### Gene annotations and *in silico* gene expression analysis

Stable-associated SNPs were blasted on whole genome shotgun contigs of *B. juncea* var. Varuna using the NCBI blast tool. The genomic region of 1.05 Mb flanking (upstream and downstream) the SNP position has been used and subjected to gene prediction using the online tool Softberry FGENESH [61]. The length of the region flanking the SNPs was taken as the value of linkage-disequilibrium decay (LD decay) in the current experiment, i.e.*,* 1.05 Mb. Further, these predicted genes of *B. juncea* were annotated using *B. napus* var. De Ae genome (NCBI GENBANK) as a reference. The orthologs of annotated candidate genes were identified in the *Arabidopsis* genome using online tool Ortho DB [62] and these genes were subjected to *in silico* gene expression analysis using Klepikova *Arabidopsis* Atlas eFB Browser [63]. Subsequently, the putative function of candidate genes was predicted through literature search and significant *in silico* expression in the concerned tissues.

## Results

### Meteorological observations during *rabi* 2020–21 and 2021–22

**Fig 1:**
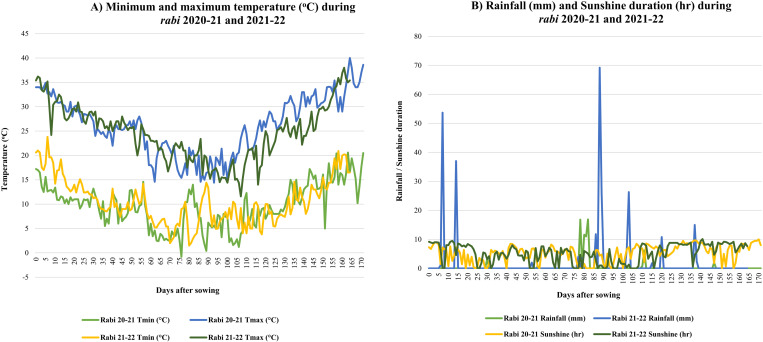
Meteorological variation observed during rabi 2020-21 and 2021–22; A) minimum and maximum temperature range, B) Rainfall pattern and Sunshine duration.

Observations on weather parameters have provided the extent of meteorological variations observed during *rabi* 2020–21 and 2021–22 (Fig 1). A higher range of minimum and maximum temperatures was observed during *rabi* 2021–22, with a value of 1.5 to 23.8 °C and 11.6 to 38 °C, respectively; compared to that of *rabi* 2020–21. Similarly, higher rainfall was observed during *rabi* 2021–22 (mean = 1.66 mm and range = 0.00 to 69.2 mm) compared to *rabi* 2020–21 (mean = 0.40 mm and range = 0.00 to 16.9 mm). In *rabi* 2020–21, significant amount of rainfall was observed during 78–82 days after sowing (DAS; siliqua development stage), while in *rabi* 2021–22, it was more prominent during early germination (7 DAS), early vegetative (14 DAS), siliqua development (86–90 DAS; 103 DAS) and maturity phase (120 DAS). Conversely, mean sunshine duration was observed in *rabi* 2020–21 (5.34 hr), compared to *rabi* 2021–22 (5.23 hr) (S3 Table).

### Phenotypic evaluation of GWAS panel for agro-morphological and oil quality attributes

Season-wise (*Rabi*, 2020–21 and *Rabi*, 2021–22) analysis of variance (ANOVA) revealed significant differences among the genotypes for all of the studied traits (Table 1). It shows the existence of substantial variation among genotypes. In *rabi* 2020–21, higher mean values were observed for DF (53.82 days), DFT (98.00 days), MSL (79.12 cm), PBPP (4.62), SBPP (13.18), SL (3.67 cm), SYPP (18.55 g), BYPP (79.86 g), OC (38.40%), OA (23.70%), EcA (4.95%), and ErA (23.32%), while for *rabi* 2021–22, it was higher for DM (143.17 days), PH (234.04 cm), SPP (427.51), SPS (14.21), Glu (81.20 µmol/g), PA (9.40%), LliA (30.25%), and LlnA (15.27%). The summary statistics of the germplasm panel have shown a wide range for the evaluated traits suggesting greater diversity in the GWAS panel (Table 1). In *rabi* 2020–21, a wider range was observed for DF (38.68–112.39 days), DM (126.82–171.11 days), PH (141.25–295.59 cm), MSL (28.93–118.13 cm), PBPP (1.99–12.27), SBPP (7.18–34.95), BYPP (28.43–166.06 g), OC (28.89-46.13%), EcA (0.4-10.37%) and ErA (0.72–40.04%), while remaining traits such as DFT (65.61–143.04 days), SPP (201.45–693.99), SL (1.85–5.39 cm), SPS (8.08–19.93), SYPP (3.22–35.10 g), Glu (8.43–132.90 µmol/g), PA (5.53–14.99%), OA (7.98–47.09%), LliA (21.55–46.46%), and LlnA (9.76–23.40%) have shown a higher range in *rabi* 2021–22.

**Table 1:** Descriptive statistics of GWAS panel for different agro-morphological and quality traits evaluated during *Rabi*, 2020-21 and *Rabi*, 2021–22.

Traits	Genotypic MSS		CV		Mean		Range	
***Rabi* 20–21**	***Rabi* 21–22**	***Rabi* 20–21**	***Rabi* 21–22**	***Rabi* 20–21**	***Rabi* 21–22**	***Rabi* 20–21**	***Rabi* 21–22**
DF	140.23 ^**^	113.60 ^**^	3.41	4.61	53.82	51.99	38.68–112.39	35.07–99.79
DFT	142.50 ^**^	198.28 ^**^	4.05	6.59	98.00	91.08	81.39–138.54	65.61–143.04
DM	48.79 ^**^	52.80 ^**^	2.29	2.21	141.04	143.17	126.82–171.11	121.39–164.39
PH	811.02 ^**^	656.31 ^**^	4.70	5.20	214.97	234.04	141.25–295.59	167.04–293.94
MSL	267.06 ^**^	256.80 ^**^	7.99	8.99	79.12	76.86	28.93–118.13	30.24–113.81
PBPP	2.38 ^**^	1.09 ^**^	11.08	11.02	4.62	4.61	1.99–12.27	2.98–9.52
SBPP	14.17 ^**^	9.22 ^**^	11.11	10.37	13.18	13.06	7.18–34.95	5.48–21.54
SPP	11739.22 ^**^	10741.54 ^**^	15.33	13.90	398.78	427.51	172.21–644.99	201.45–693.99
SL	0.48 ^**^	0.58 ^**^	6.54	8.64	3.67	3.58	2.37–5.16	1.85–5.39
SPS	2.85 ^*^	3.62^*^	7.50	9.33	14.16	14.21	9.57–18.56	8.08–19.93
SYPP	40.21 ^**^	47.20^**^	15.63	16.13	18.55	16.92	4.89–36.14	3.22–35.1
BYPP	582.27 ^**^	660.87 ^**^	14.27	14.82	79.86	79.65	28.43–166.06	39.7–151.29
OC	10.29 ^**^	8.01 ^**^	3.59	3.30	38.40	37.30	28.89–46.13	29.65–43.82
Glu	536.64 ^**^	658.94 ^**^	10.24	10.02	76.37	81.20	7.58–126.64	8.43–132.9
PA	2.61 ^**^	4.04 ^**^	7.36	9.29	7.72	9.40	3.81–11.95	5.53–14.99
OA	86.07 ^**^	65.88 ^**^	6.04	7.38	23.70	23.19	12.96–49.15	7.98–47.09
LliA	21.11 ^**^	19.79 ^**^	8.71	6.45	26.95	30.25	19.09–41.25	21.55–46.46
LlnA	5.24 ^**^	7.70 ^**^	7.53	6.17	13.28	15.27	8.73–21.56	9.76–23.4
EcA	4.50 ^**^	4.65 ^**^	10.05	10.00	4.95	3.61	0.4–10.37	0.2–9.31
ErA	113.53 ^**^	88.79 ^**^	7.65	8.51	23.32	18.59	0.72–40.04	0.3–35.21

DF = Days to 50% flowering, DFT = Days to 100% flower termination, DM = Days to maturity, PH = Plant height, MSL = Main shoot length, PBPP = Primary branches per plant, SBPP = Secondary branches per plant, SPP = Siliquae per plant, SL = Siliqua length, SPS = Seeds per siliqua, SYPP = Seed yield per plant, BYPP = Biological yield per plant, OC = Oil content, Glu = Glucosinolates, PA = Palmitic acid, OA = Oleic acid, LliA = Linoleic acid, LlnA = Linolenic acid, EcA = Eicosenoic acid, ErA = Erucic acid, Genotypic MSS = Genotypic mean sum of squares, CV = Coefficient of variation

‘

*’,’

**’ indicate significant difference at 5% and 1% level of significance, respectively.

**Fig 2:**
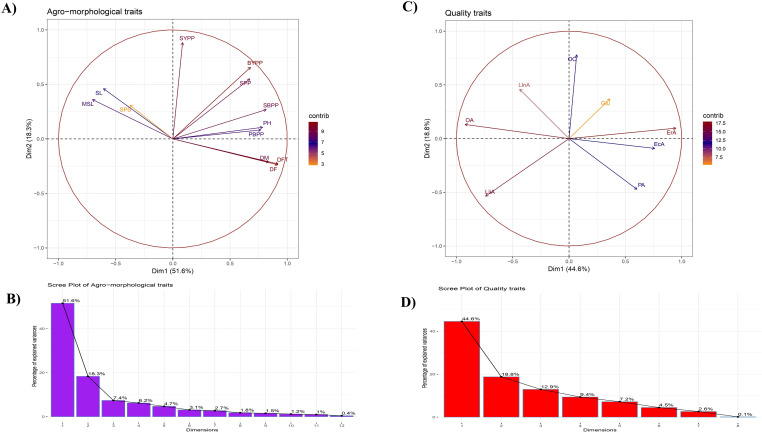
Principal component analysis (PCA); (A) PCA biplot of agro-morphological attributes, (B) Scree plot of agro-morphological traits showing total variation explained by individual PCs, (C) PCA biplot of quality attributes, (D) Scree plot of quality parameters showing total variation explained by individual PCs.

Furthermore, PCA of agro-morphological and oil quality attributes gave an insight into a better understanding of phenotypic variation. For agro-morphological traits, first two principal components, PC1 (51.6%) and PC2 (18.3%) cumulatively explain 69.9% of the total variation, (Fig 2A, Fig 2B). It has been observed that DF, DFT, DM, PH, MSL, PBPP, and SBPP have more contribution to PC1, while SYPP has more contribution to PC2. Interestingly, SPP, SL, SPS, and BYPP have considerable contributions to both PCs. In PC1, most of the traits (DF, DFT, DM, PH, PBPP, SBPP, SPP, SYPP, and BYPP) contributed positively, while MSL, SL, and SPS contributed negatively. Similarly, in PC2, PH, MSL, PBPP, SBPP, SPP, SL, SPS, SYPP, and BYPP made positive contribution, whereas flowering traits (DF, DFT, and DM) contributed negatively (Fig 2A). However, for oil quality attributes, first two PCs cumulatively explained 63.4% total variation, with individual contribution of 44.6 and 18.8% by PC1 and PC2, respectively (Fig 2C, Fig 2D). Individual fatty acids, e.g.*,* OA, EcA, and ErA had more contribution towards PC1, while OC had more contribution to PC2. Moreover, the remaining traits including Glu, PA, LliA, and LlnA exhibited remarkable contribution to both PCs. The contribution of most of the variables was positive to PC1 except for OA, LliA, and LlnA, whereas, only PA, LliA, and EcA contributed negatively to PC2 (Fig 2C). Directions of eigenvectors represented the correlation among variables. In the current study, SYPP was positively correlated with MSL, SBPP, SPP, SL, and BYPP. Flowering traits (DF, DFT, and DM) were positively correlated with each other. Conversely, quality parameters such as ErA was positively correlated with PA, EcA, and Glu, whereas negative correlation was observed with OA, LliA, and LlnA (Fig 2).

### Population structure, diversity, analysis of molecular variance and LD decay of GWAS panel

**Fig 3:**
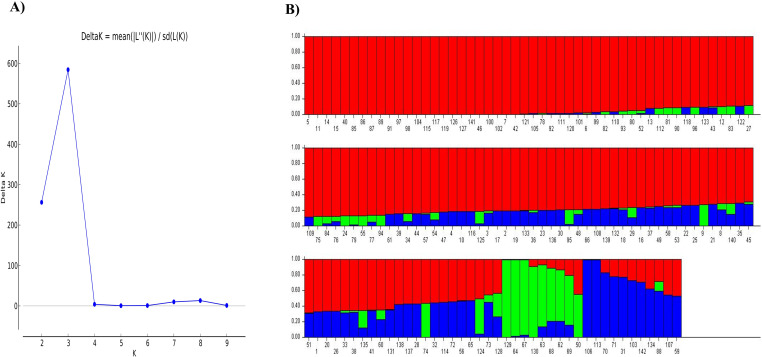
Population structure analysis; (A) Delta-K value to optimize number of sub-populations, (B) Grouping of GWAS panel based on sub-populations.

**Fig 4:**
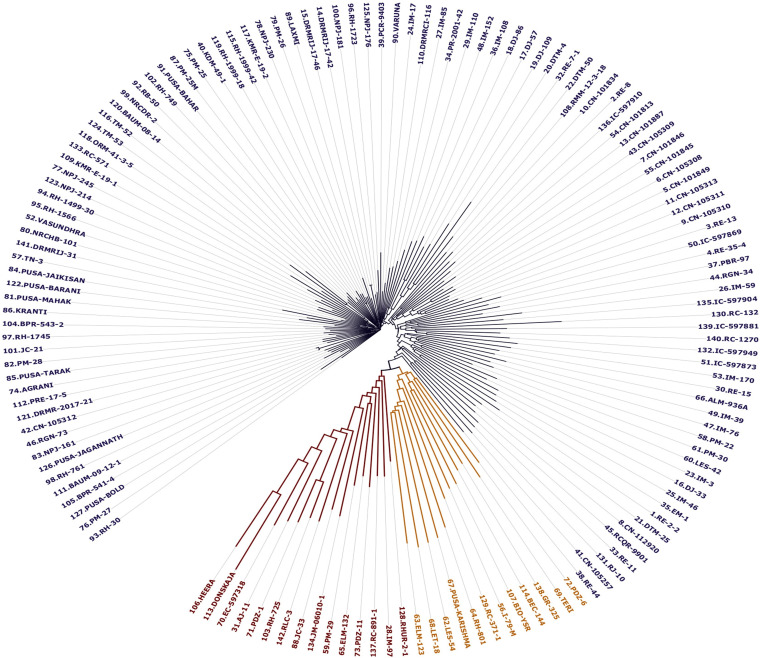
Neighbour joining diversity tree showing molecular diversity among GWAS panel. Genotypes highlighted with different colours denoting different clusters.

**Fig 5:**
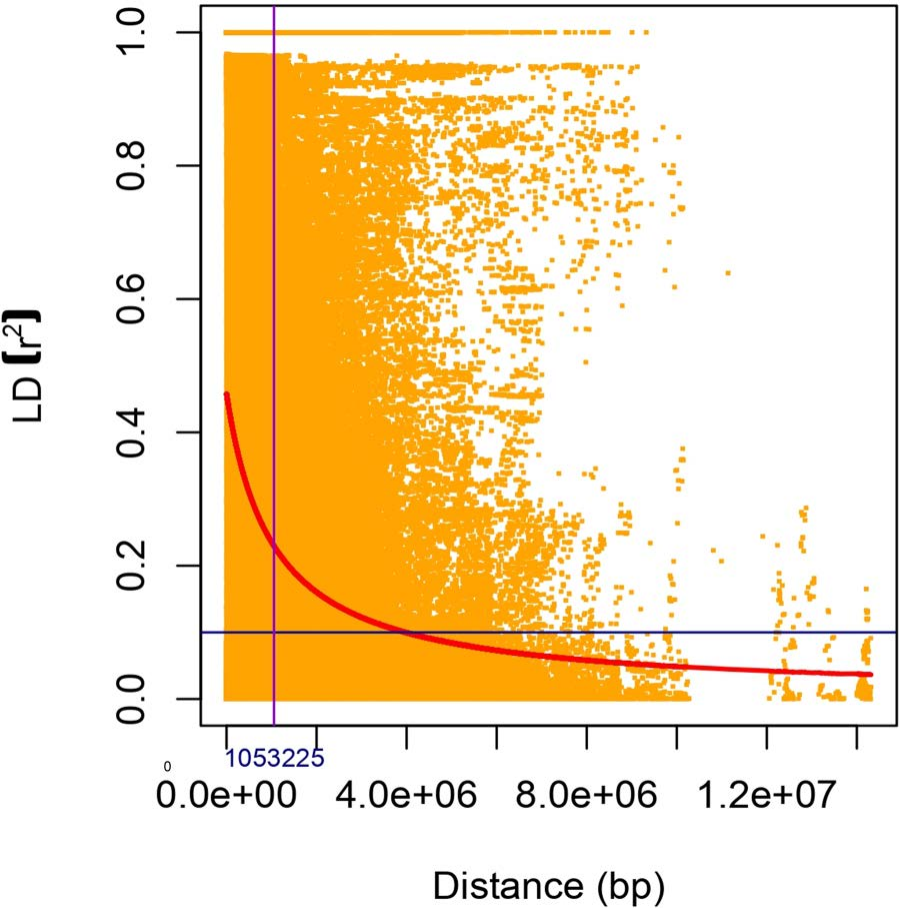
LD decay plot of GWAS panel using SNP markers.

The allele admixture model of structure analysis exhibited maximum ∆k value at k = 3, indicating GWAS panel consists of three sub-populations (SP; Fig 3A, Fig 3B). SP1, SP2, and SP3 comprised 121, 9, and 12 genotypes, with respective allelic contributions of 73.80%, 8.60%, and 17.60% to the whole germplasm panel (Table 2). SP1 comprised most of the conventional genotypes, which included both exotic and indigenous types. Whereas, SP2 mainly composed of Indian germplasm (IC-597869, RC-371-1, and RC-132) and quality mustard (LES-54, ELM-123, RH-801, Pusa Karishma, LET-18, and TERI). Similarly, SP3 consists of Australian germplasm (AJ-11, JM-06010–1), quality mustard (PM-29, EC-597318, PDZ-1/PDZM-31, PDZ-11/PDZM-33, JC-33, and RLC-3) and European genotypes (Donskaza and Heera), etc. Moreover, the maximum average distance was observed in SP2 (0.59), whereas, it was least in SP1 (0.17). In contrast, the Fixation Index (*Fst*) was maximum in SP1 (0.76) and was lowest in the case of SP2 (0.15; Table 2). In the GWAS panel, 24 conventional genotypes of SP1, one Indian genotype (RC-371-1) of SP2, and two European genotypes (Heera and Donskaza) of SP3 did not have the allele admixtures while the rest of the genotypes contain allele admixtures (*alleles* from more than one sub-population). The population structure analysis provided information about the molecular relationship among genotypes. To partition total genotypic variation into within and among sub-populations molecular variance (AMOVA) analysis was performed, wherein 36.95% variation was observed among populations, and the remaining variation (63.05%) was attributed to within sub-populations (Table 2). Furthermore, to get a better insight into molecular diversity, a tree-based diversity analysis of the GWAS panel *via* neighbor-joining method was performed. It was observed that genotypes belonging to the same sub-population were more closely related; whereas, genotypes belonging to different sub-populations were more diverse. In diversity analysis, three major clusters were detected as observed in structure analysis (Fig 4). In the current study, the LD decay analysis revealed that up to 1.05 Mb (1053225 bp), the LD value persists, after which it begins to decay (Fig 5). Markers located within a physical distance of less than 1.05 Mb tended to be in LD; therefore, this region was utilized for gene annotation of stable Marker-Trait Associations (MTAs).

**Table 2:** Analysis of molecular variance and population structure analysis.

Source	Df	SS	MSS	Estimated Variance	% contribution
Among populations	2	5752.39	2876.20	141.26	36.95
Within populations	139	33507.95	241.06	241.06	63.05
Total	141	39260.34	278.44	382.32	100
**Sub-populations**	**Number of individuals**	**Average distance (He)**	**Fst**	**Overall proportion**	
SP1	121	0.17	0.76	73.80%	
SP2	09	0.59	0.15	8.60%	
SP3	12	0.28	0.65	17.60%	

Df = Degrees of freedom, SS = Sum of squares, MSS = Mean sum of squares, % = Percent contribution of total variance

### Marker-trait associations (MTAs) of agro-morphological and quality attributes

**Fig 6:**
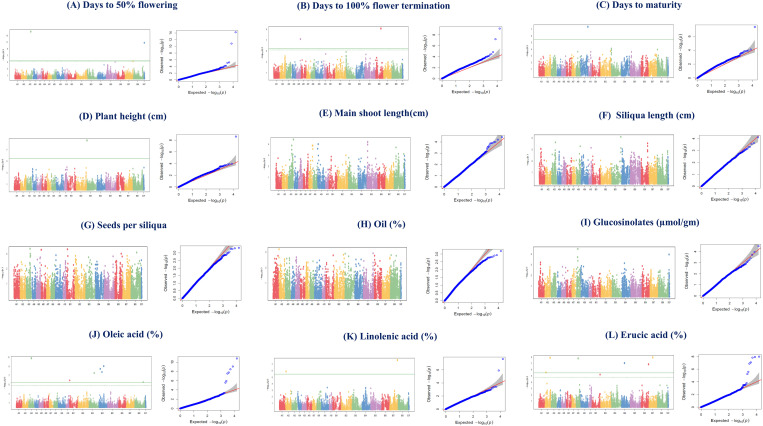
Manhattan and QQ plots showing MTAs for different agro-morphological traits during *rabi* 2020-21.

**Fig 7:**
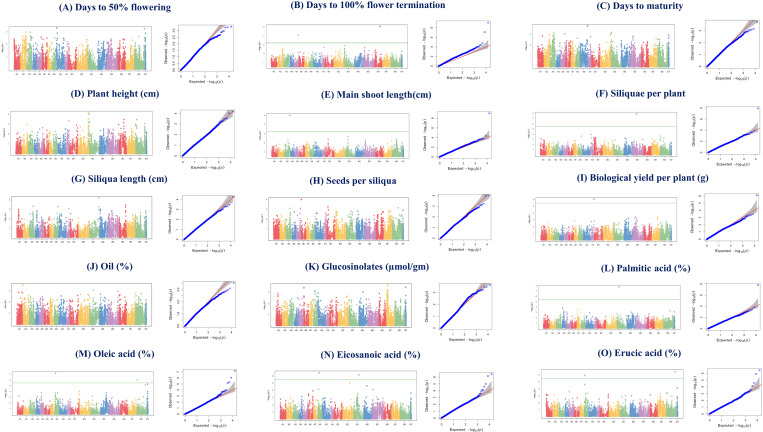
Manhattan and QQ plots showing MTAs for different agro-morphological traits during *rabi* 2021–22.

GWAS analysis was conducted using the BLINK model in the R-based package GAPIT on various agro-morphological and oil quality attributes. A total of 49 SNPs were identified, with 28 and 21 SNPs detected in the *rabi* seasons of 2020–21 and 2021–22, respectively (Table 3, Fig 6 and 7). Among these, 16 and 9 SNPs were specific to the *rabi* 2020–21 and 2021–22, respectively, while the remaining 12 SNPs were common to both seasons and were utilized for further analysis. In the *rabi* 2020–21, two MTAs were identified for each of DF (Bn-A03-p19798208 and Bn-scaff_18360_1), DFT (Bc-B6-p26812452 and Bn-A05-p21930978), SL (Bn-A03-p21210189 and Bn-A08-p19365594), SPS (Bn-A03-p21615990 and Bn-A06-p2234696), and LInA (Bj-B7-p38672208 and Bn-A02-p3300731). Conversely, single SNPs were associated with traits including DM (Bn-A09-p36455112), PH (Bn-A09-p36455112), MSL (Bn-A03-p28109103), OC (Bn-A02-p636428), and Glu (Bn-A08-p8658074). Additionally, traits such as OA exhibited seven MTAs (Bn-A03-p15957657, Bj-B4-p25774828, Bj-B4-p9000035, Bj-B4-p17202410, Bj-B3-p25944867, Bj-B1-p2664020, and Bj-B8-p41554126), while ErA displayed six (Bj-B7-p19658501, Bn-A02-p25655528, Bn-A08-p10822662, Bj-B4-p17190386, Bj-B6-p37724780, and Bn-A02-p5612147). In *rabi* 2020–21, two SNPs were identified for each of DFT (Bc-B6-p26812452 and Bn-A05-p21930978), SL (Bn-A03-p21210189 and Bn-A08-p19365594), SPS (Bn-A03-p21615990 and Bn-A06-p2234696), OA (Bn-A08-p4254041 and Bj-B8-p3030447), EcA (Bn-A08-p7563202 and Bj-B3-p25039645) and ErA (Bj-B8-p41554126 and Bn-A08-p4001683), while single SNPs were found for DF (Bn-A03-p19798208), DM (Bn-A09-p36455112), PH (Bc-B3-p317964), MSL (Bn-A03-p28109103), SPP (Bj-B5-p19074770), BYPP (Bn-A10-p15838932), OC (Bn-A02-p636428), Glu (Bn-A08-p8658074), and PA (Bj-B3-p9090824) (Table 3).

**Table 3:** List of marker-trait associations identified during *Rabi*, 2020-21 and *Rabi*, 2021–22.

Season	Trait	SNP	Allele	Chrom	Position	*p*-value	MAF	Effect	PVE%
***Rabi*, 2020–21**	DF	Bn-A03-p19798208	A/C	A03	19798208	6.08 x 10^–15^	0.15	7.90	21.24
		Bn-scaff_18360_1	A/C	NA	1296271	1.42 x 10^–11^	0.09	7.46	27
	DFT	Bc-B6-p26812452	T/C	B06	26812452	7.12 x 10^–10^	0.12	−7.83	20.27
		Bn-A05-p21930978	A/G	A05	21930978	6.43 x 10^–08^	0.23	−4.89	15.76
	DM	Bn-A09-p36455112	T/C	A09	36455112	4.31 x 10^–08^	0.10	3.10	48.89
	PH	Bc-B3-p317964	A/C	B03	317964	2.40 x 10^–09^	0.23	11.89	29.02
	MSL	Bn-A03-p28109103	A/G	A03	28109103	0.00004	0.10	10.17	NA
	SL	Bn-A03-p21210189	A/C	A03	21210189	0.0002	0.11	−0.35	NA
		Bn-A08-p19365594	A/G	A08	19365594	0.0005	0.15	−0.29	NA
	SPS	Bn-A03-p21615990	A/C	A03	21615990	0.0005	0.15	−0.66	NA
		Bn-A06-p2234696	A/G	A06	2234696	0.0005	0.12	0.70	NA
	OC	Bn-A02-p636428	T/G	A02	636428	0.0006	0.50	−0.43	NA
	Glu	Bn-A08-p8658074	T/C	A08	8658074	0.0004	0.10	−12.22	NA
	OA	Bn-A03-p15957657	T/G	A03	15957657	1.57 x 10^–11^	0.10	6.85	15.14
		Bj-B4-p25774828	A/G	B04	25774828	8.57 x 10^–10^	0.24	3.55	4.85
		Bj-B4-p9000035	T/G	B04	9000035	3.49 x 10^–09^	0.21	3.29	4.39
		Bj-B4-p17202410	A/G	B04	17202410	1.70 x 10^–08^	0.12	4.44	7.7
		Bj-B3-p25944867	A/G	B03	25944867	2.85 x 10^–08^	0.18	4.37	6.04
		Bj-B1-p2664020	T/C	B01	2664020	1.17 x 10^–06^	0.42	−2.10	1.53
		Bj-B8-p41554126	A/G	B08	41554126	2.63 x 10^–06^	0.14	−2.86	3.33
	LlnA	Bj-B7-p38672208	A/G	B07	38672208	2.22 x 10^–08^	0.27	−1.05	11.6
		Bn-A02-p3300731	A/C	A02	3300731	1.25 x 10^–06^	0.08	1.36	27.23
	ErA	Bj-B7-p19658501	A/G	B07	19658501	1.06 x 10^–08^	0.23	5.16	7.98
		Bn-A02-p25655528	A/C	A02	25655528	1.21 x 10^–08^	0.14	−4.39	9
		Bn-A08-p10822662	A/C	A08	10822662	1.51 x 10^–08^	0.26	−3.86	5.87
		Bj-B4-p17190386	T/C	B04	17190386	8.60 x 10^–08^	0.12	−4.22	8.27
		Bj-B6-p37724780	T/G	B06	37724780	1.35 x 10^–07^	0.09	4.06	6.67
		Bn-A02-p5612147	T/C	A02	5612147	2.68 x 10^–06^	0.11	−3.75	5.4
***Rabi*, 2021–22**	DF	Bn-A03-p19798208	A/C	A03	19798208	0.001	0.15	5.19	NA
	DFT	Bc-B6-p26812452	T/C	B06	26812452	8.18 x 10^–10^	0.12	−7.86	20.15
		Bn-A05-p21930978	A/G	A05	21930978	7.88 x 10^–08^	0.23	−4.89	15.51
	DM	Bn-A09-p36455112	T/C	A09	36455112	0.0002	0.10	2.83	NA
	PH	Bc-B3-p317964	A/C	B03	317964	0.0001	0.23	10.27	NA
	MSL	Bn-A03-p28109103	A/G	A03	28109103	9.68 x 10^–10^	0.10	11.97	50.22
	SPP	Bj-B5-p19074770	T/C	B05	19074770	1.21 x 10^–08^	0.17	−39.12	35.37
	SL	Bn-A03-p21210189	A/C	A03	21210189	0.0005	0.11	−0.34	NA
		Bn-A08-p19365594	A/G	A08	19365594	0.0005	0.15	−0.30	NA
	SPS	Bn-A03-p21615990	A/C	A03	21615990	0.0008	0.15	−0.64	NA
		Bn-A06-p2234696	A/G	A06	2234696	0.0001	0.12	0.79	NA
	BYPP	Bn-A10-p15838932	T/G	A10	15838932	8.55 x 10^–07^	0.44	−6.67	12.96
	OC	Bn-A02-p636428	T/G	A02	636428	0.0003	0.50	−0.44	NA
	Glu	Bn-A08-p8658074	T/C	A08	8658074	0.0009	0.10	−11.45	NA
	PA	Bj-B3-p9090824	A/G	B03	9090824	1.50 x 10^–08^	0.24	−0.89	31.61
	OA	Bn-A08-p4254041	T/G	A08	4254041	6.78 x 10^–08^	0.12	−2.83	36.83
		Bj-B8-p3030447	T/C	B08	3030447	8.76 x 10^–07^	0.15	−3.72	19.95
	EcA	Bn-A08-p7563202	T/C	A08	7563202	3.69 x 10^–07^	0.11	1.37	17.46
		Bj-B3-p25039645	A/C	B03	25039645	7.00 x 10^–07^	0.15	0.98	14.08
	ErA	Bj-B8-p41554126	A/G	B08	41554126	3.34 x 10^–07^	0.14	3.62	29.99
		Bn-A08-p4001683	T/G	A08	4001683	1.16 x 10^–06^	0.16	−5.30	1.3

Chrom = Chromosome, MAF = Minor allele frequency, PVE% = Percent phenotypic variation explained

### Gene annotations of nearby regions of stable SNPs and *in silico* expression analysis of candidate genes

Among 49 associated SNPs, a total of 12 SNPs were consistent in both seasons for studied traits. Ten SNPs have shown association with 7 agro-morphological traits *viz*., DF, DFT, DM, PH, MSL, SL, and SPS, and two stable MTAs were recorded for 2 quality attributes, i.e.*,* OC and Glu. These SNPs were considered stable MTAs and used for further analysis. In the present study, gene annotations of stable SNPs considering the LD identified several candidate genes for studied traits (Table 4). For instance, in DF, SNP Bn-A03-p19798208 was associated with three significant candidate genes, including *BTB/POZ domain-containing protein*, *WRKY transcription factor 1*, and *Heading date 3b*, which exhibited notably higher *in silico* expression in flowering tissues, indicating their crucial role in flowering regulation. (Table 4). Similarly, DFT revealed two stable SNPs, Bn-A05-p21930978 and Bc-B6-p26812452, closely located with 5 (*Photoperiod-independent early flowering 1, agamous-like MADS-box protein AP1, FBD-associated F-box protein, REVEILLE 8* and *FAR1-related sequence 5*) and 4 (*Ethylene-responsive transcription factor 9, auxin-responsive protein IAA10, pectate lyase 1 and ethylene response sensor 2*) candidate genes, respectively, showing maximum expression either in flowering or mature tissues as identified through *in silico* analysis. Moreover, stable SNP Bn-A09-p36455112 of DM was closely linked with 5 candidate genes (*Sialyltransferase-like protein 1, early nodulin-like protein 1, axial regulator YABBY 2, ethylene-responsive transcription factor ERF023,* and e*thylene-responsive transcription factor 1*). The *in-silico* analysis revealed that these were highly expressive in flowering and later stages of the plant, suggesting the putative role in plant maturity. PH-associated SNP Bc-B3-p317964 had two linked genes, *IAA17* and *B-box zinc finger protein 24*, specifically showing higher expression at seedling hypocotyl and senescent internode, respectively. In the case of MSL, SNP Bn-A03-p28109103 harbored 3 candidate genes *viz.*, *cup-shaped cotyledon 3, SMAX1,* and *short hypocotyl in white light 1*, predominantly expressing in terminal and mature tissues of the plant identified through *in silico* expression analysis. Additionally, SL exhibited two stable associations, Bn-A03-p21210189 and Bn-A08-p19365594, linked to *GATA transcription factor 25* and *E3 ubiquitin-protein ligase BOI*, respectively, with significant *in silico* gene expression at pod of the senescent silique and carpels of the young flower, respectively. Conversely, SNPs Bn-A03-p21615990 and Bn-A06-p2234696 were consistently associated with SPS, closely linked with three candidate genes (*Nuclear fusion defective 4*, *GATA transcription factor 25*, *APRR1*) and one candidate gene (*GATA transcription factor 11*), respectively, within the LD block, showing significant *in silico* expression in seed-bearing tissues such as ovules and siliqua. Furthermore, two stable MTAs were observed for quality attributes, one each for OC and Glu. OC was associated with SNP Bn-A02-p636428 carrying candidate genes *WRI1* and *Oleosin*, exhibiting higher *in silico* expression in seeds. Similarly, the candidate gene, *Transcription factor MYB123-1.1* linked to Glu MTA Bn-A08-p8658074 has also shown maximum expression in seeds (Table 4).

**Table 4:** . Gene annotations of stable SNPs and *in silico* tissue expression analysis of identified candidate genes.

Traits	Associated SNPs	FA	AA	Candidate genes	Gene ID	*A. thaliana* Orthologs	Expressing Tissues	Expression level	Control	Fold-Change
DF	Bn-A03-p19798208	A	C	*BTB/POZ domain-containing protein*	111214206	AT2G04740	Flowers	7.28	5.23	1.39
				*WRKY transcription factor 1*	106426741	AT5G35750	Axis of inflorescence	8.79	5.17	1.7
				*Heading date 3b*	106439656	AT3G21320	Anthers of the mature flower	21.76	0.02	1279.91
DFT	Bn-A05-p21930978	G	A	*Photoperiod-independent early flowering 1*	106452362	AT3G12810	Carpels of the young flowers	10.24	4.39	2.33
				*Agamous-like MADS-box protein AP1*	106412766	AT1G69120	Sepals of the young flower	99.08	0.03	3416.62
				*FBD-associated F-box protein*	125608703	AT1G05080	Anthers of the young flower	5.9	0.02	245.82
				*REVEILLE-8*	111215777	AT3G09600	Opened anthers	16.43	0.45	36.19
				*FAR1-related sequence 5*	106452929	AT3G07500	Senescent silique	12.98	1.75	7.42
	Bc-B6-p26812452	T	C	*Ethylene-responsive transcription factor 9*	106398162	AT1G03800	Young seeds	1.14	0.2	5.63
				*Auxin-responsive protein IAA10*	106402229	AT1G04100	Pod of the senescent silique	5.99	1.49	4.01
				*Pectate lyase 1*	106370994	AT1G04680	Petals of the mature flower	107.66	16.98	6.34
				*Ethylene response sensor 2*	106371023	AT1G04310	Petals of the mature flower	18.78	4.96	3.79
DM	Bn-A09-p36455112	T	C	*Sialyltransferase-like protein 1*	106420237	AT1G08660	Flowers	20.98	11.05	1.9
				*Early nodulin-like protein 1*	106419837	AT1G08500	Petals of the mature flower	43.06	1.4	30.69
				*Axial regulator YABBY 2*	106419835	AT1G08465	Sepals of the mature flower	21.91	4.05	5.41
				*Ethylene-responsive transcription factor ERF023*	106381811	AT1G01250	Petals of the mature flower	40.05	2.5	16.04
				*Ethylene-responsive transcription factor 1*	106434150	AT4G17500	Leaf Petiole of the senescent leaf	273.82	17.04	16.07
PH	Bc-B3-p317964	A	C	*Auxin-responsive protein IAA17*	125591576	AT1G04250	Seedling Hypocotyl	31.58	3.61	8.74
				*B-box zinc finger protein 24*	106365696	AT1G06040	Senescent internode	67.59	28.07	2.41
MSL	Bn-A03-p28109103	G	A	*Cup-shaped cotyledon 3*	106386092	AT4G28530	Senescent internode	8.92	0.23	39.65
				*SMAX1*	106441046	AT4G30350	Sepals of the young flower	62.25	16.04	3.88
				*Short hypocotyl in white light 1*	106441155	AT4G33780	Stigmatic tissue	43.57	10.24	4.25
SL	Bn-A03-p21210189	A	C	*GATA transcription factor 25*	106439837	AT4G24470	Pod of the senescent silique	10.98	9.15	1.2
	Bn-A08-p19365594	A	G	*E3 ubiquitin-protein ligase BOI*	106362423	AT1G10650	Carpels of the young flower	12.01	7.11	1.69
SPS	Bn-A03-p21615990	A	C	*Nuclear fusion defective 4*	106439838	AT4G34950	Silique	30.45	6.88	4.43
				*GATA transcription factor 25*	106439837	AT4G24470	Ovules	13.0	9.15	1.42
				*Two-component response regulator APRR1*	125607080	AT5G61380	Pod of the senescent silique	63.88	36.04	1.77
	Bn-A06-p2234696	G	A	*GATA transcription factor 11*	106346369	AT1G08010	Ovules	20.5	9.49	2.16
OC	Bn-A02-p636428	T	G	*Ethylene-responsive transcription factor WRI1*	106352859	AT3G54320	Seeds	32.34	2.45	13.19
				*Oleosin*	106352688	At5g07260	Seeds	32.29	0.01	2152.93
Glu	Bn-A08-p8658074	C	T	*Transcription factorMYB123-1.1*	106418890	AT5G35550	Seeds	60.06	0.14	444.88

FA = Favourable allele, AA = Alternate allele

## Discussion

GWAS panel in the present investigation comprises of advanced breeding lines, introgression lines (ILs), released varieties, and indigenous as well as exotic collections. Analysis of variance (ANOVA) is the initial step of any experimental design to determine the significance of treatments under study. In the present study, the significant genotypic variation for all the studied traits suggested that the genotype collection carries vast genetic variability, which could be efficiently utilized in the GWAS. Germplasm accessions having wide variability are considered as a potential source for genetic improvement. Hence, enhancing quantitative traits like agro-morphological and quality attributes require the identification of genetic variations present in the germplasm, followed by their utilization in breeding programs [39]. In the present study, the *rabi* 2020–21 demonstrated superior mean performance and a wide range for most of the studied traits as compared to *rabi* 2021–22, indicating the positive influence of its meteorological parameters on the trait performance. Conversely, the *rabi* 2021–22 experienced a higher temperature range, irregular and frequent rainfall during later growth stages, and reduced sunshine duration, rendering it unfavourable for most of the studied traits. PCA is regarded as an effective method for extracting key information from phenotypically complex traits with high correlations, while preserving the original data integrity [64]. In the present investigation, major variation was explained by the first two PCs (PC1 and PC2) for agro-morphological and quality parameters, suggesting the efficiency of PCA in data visualization by dimension reduction.

The advancement of molecular markers has facilitated the application of various mapping approaches for diverse traits across plant species. For instance, these approaches have been used for mapping micronutrients, grain quality, and agronomic traits[65], abiotic stress tolerance [66,67], and grain yield components [68] in bread wheat. Similarly, ionome-related QTLs have been mapped in *A. thaliana* [69], and candidate genes for phenolic acids and flavonoids [70], as well as agronomic and yield-related traits under drought stress [71] have been identified in rapeseed. Moreover, SNP genotyping has revolutionized molecular breeding, as it is widely used for its efficiency and precision [15]. The array-based genotyping preferred over GBS due to their reliability, fixed loci, and simpler analysis [16,17]. Therefore, in the current study, genome-wide association study (GWAS) has been undertaken using 90K *Brassica* array-based genotyping. These SNPs were widely distributed throughout the genome of *B. juncea*. Structure analysis using STRUCTURE software for 142 genotypes of *B. juncea* showed that the GWAS panel consisted of three sub-populations. Diversity analysis using the neighbor-joining method also suggested three major clusters, confirming the three sub-populations in the GWAS panel. It is important to note that genotypes that belong to the same sub-populations were more closely related in diversity analysis as compared to those that occur in different sub-populations. Genotypic diversity and structure analysis have provided the evolutionary significance of genotypes. For instance, genotypes belonging to group-I were mostly of Indian origin and conventional type. Group-II genotypes were intermediate type, mostly quality mustard, that shares genes from both Indian and exotic germplasms (Australian and European). However, the third group contains European germplasms and Indian-quality genotypes derived from European mustard, containing the majority of genes from European parents. Moreover, the allele admixture as observed in the present study suggests the gene flow among the sub-populations. Therefore, this diverse panel can provide more valuable implications as compared to bi-parental populations, by utilizing maximum allelic diversity and historic recombination [72].

The total genotypic variance observed in the GWAS panel was partitioned using AMOVA, which has more within-sub-population variation compared to among sub-populations, which indicated the existence of sufficient diversity within a sub-population. *Fst* values give the idea of the degree of differentiation among populations [73]. The *Fst* values of SP1(0.76) and SP3 (0.65) have shown very strong differentiation, while SP2 (0.15) has shown moderate differentiation from the original population.

The number of markers needed for association mapping depends on the rate of LD decay, which is determined by the genetic distance between markers [74]. It has been observed that the mode of pollination affects the LD decay rate, i.e.*,* self-pollinating crops have longer LD blocks and decay slower compared to cross-pollinating crops. For instance, wheat exhibited LD decay at a distance of 7.15 Mb [75], whereas maize decayed at a distance of about 2 kb [76]. Similarly, in this study, moderate LD decay (1.05 Mb) was observed in *B. juncea*, as the crop has shown considerable cross-pollination based on bee activities. This region was subsequently used for gene annotations. Similarly, Vos et al. [77] suggested that LD decay can be used to determine the size of a candidate gene region.

To identify genomic regions for quantitative traits, genome-wide association studies (GWAS) is considered a powerful approach [21]. The season-wise GWAS detected 28 MTAs detected during *rabi*, 2020–21 and 21 MTAs during *rabi*, 2021–22 for various agro-morphological and quality attributes. Some MTAs were specific to only one season, indicating highly influenced by the environment. Such SNPs would not be reproducible; thus, cannot be used in the MAB. It has been observed that the threshold for GWAS using Bonferroni-correction is too stringent which leads to false negatives [78]. Therefore, we adapted less stringent criteria (p<0.001), similar to those utilized by Devate et al. [75] and Mroz et al. [60], for the identification of stable MTAs. Interestingly, as per our assumptions, when consistently occurring SNPs with p<0.001 were subjected to gene annotations, some remarkable genes such as *WRI1* and *Oleosin* in case of OC MTA Bn-A02-p636428, *TF MYB123-1.1* in Glu MTA Bn-A08-p8658074 etc. were identified. Therefore, if any SNP occurs consistently across the environments, but falls below the Bonferroni threshold; then, using a less stringent approach will be rewarding.

The stable SNPs when subjected to *in silico* analysis, various candidate genes were predicted. In the current study, SNP Bn-A03-p19798208 identified candidate genes for DF such as *BTB/POZ domain* family genes (*BOP* genes) delay the flowering under short-day conditions in *A. thaliana*, if present in mutant form [24]. Likewise, *WRKY transcription factors* regulates the flowering [25]. However, the third candidate gene of DF, i.e., *Hd3b*, a rice ortholog of the *EFL3* gene of *Arabidopsis,* causes delayed flowering under long-day field conditions in rice [26]. Similar to present findings, SNP Bn‐A03‐p1050893, was also located on A03 chromosome and associated with flowering time in *B. napus* [79].

Two stable MTAs (Bn-A05-p21930978 and Bc-B6-p26812452) were reported for DFT. Furthermore, Shah et al. [79] reported an SNP Bn-A05‐p2497466, located on A05 chromosome and associated with flowering time in *B. napus*, similar to the current association (Bn-A05-p21930978) on the same chromosome. Candidate genes identified for DFT have either played an important role in the regulation of flowering time [80–82], cell senescence/abscission [83] or involved in floral organ formation [84,85]. In addition to these reports, *in silico* tissue expression analysis of these genes, provided supportive evidence that these genes have significant role in the regulation of flowering in Crucifers. Therefore, these genes might be involved in various metabolic pathways and ultimately determining the flowering duration. Similar to DF and DFT, DM MTA (Bn-A09-p36455112) identified candidate genes which involved either in flowering control [86–88] or in cell senescence or programmed cell death [89]. Moreover, i*n silico* analysis revealed that these genes were highly expressive in later stages of plant development (in mature flowers and leaves), suggesting their active involvement in plant maturity. Interestingly, the involvement of some important flowering genes in DM suggests the genetic basis of positive correlation of flowering time (DF, DFT) with maturity as observed during the phenotypic screening of the germplasm panel. Candidate genes identified for PH (*IAA17* and *B-box zinc finger protein 24*) involved in the growth and development of plants [27,28]. Conversely, that of main shoot length *viz*., *cup-shaped cotyledon 3, SMAX1,* and *short hypocotyl in white light 1 were* reported to affect shoot apical meristem, photomorphogenesis and hypocotyl elongation in *Arabidopsis*, respectively [90–92]. Similar to these reports, the maximum *in silico* expression of these genes was observed in seedling hypocotyl and internodal regions, suggesting their crucial role in shoot elongation. Two stable MTAs, Bn-A03-p21210189 and Bn-A08-p19365594, were identified for SLs, associated with *GATA transcription factor 25* and *E3 ubiquitin-protein ligase BOI genes*, respectively, indicating higher *in silico* expression in siliqua developing tissues. Conversely, SPS had two stable MTAs; Bn-A03-p21615990 associated with *nuclear fusion defective 4, GATA transcription factor 25* and *APRR1* genes, and Bn-A06-p2234696 linked with *GATA transcription factor 11*, which have shown maximum *in silico* expression in seed-bearing tissues (ovules and siliqua). In support of the present investigation, Pal et al. [29] reported the candidate gene GATA15, associated with SPS, located on chromosome A06 in B. napus. Similarly, Khan et al. [38] identified two MTAs (Bn-A03-p12353370 and Bn-A03-p13109267) on A03 and one MTA (Bn-A06-p24204030) on A06, associated with SPS.

The close proximity of SL SNP Bn-A03-p21210189 and SPS SNP Bn-A03-p21615990 share a common candidate gene (*GATA transcription factor 25*) suggesting pleiotropy as the genetic basis of positive trait correlation. Additionally, stable MTAs were found for OC and Glu, with SNP Bn-A02-p636428 linked to *WRI1* and *oleosin* genes regulating oil accumulation [30,31]. Conversely, the SNP associated with Glu (Bn-A08-p8658074) had the candidate gene *Transcription factor MYB123-1.1*, known for regulating glucosinolates biosynthesis [93]. These candidate genes exhibit maximum *in silico* expression in seeds, indicating their pivotal role in determining both seed oil quantity and quality.

## Conclusion

The present study reveals substantial variability within the GWAS panel, highlighting extensive diversity across the evaluated traits. Notably, this investigation represents the first application of array-based genotyping in *Brassica juncea*, signifying a significant milestone in genetic research. The germplasm panel, comprising three distinct sub-populations as determined through structure and neighbor-joining diversity analyses, provides a valuable resource for future studies. Furthermore, this research identifies critical genomic regions influencing the inheritance of the analyzed traits, offering insights for advancing molecular breeding and genetic improvement efforts. In this study, twelve stable marker-trait associations (MTAs) were identified for key traits, including DF (Bn-A03-p19798208), DFT (Bn-A05-p21930978 and Bc-B6-p26812452), DM (Bn-A09-p36455112), PH (Bc-B3-p317964), MSL (Bn-A03-p28109103), SL (Bn-A03-p21210189 and Bn-A08-p19365594), SPS (Bn-A03-p21615990 and Bn-A06-p2234696), OIL (Bn-A02-p636428), and Glu (Bn-A08-p8658074). These MTAs harbor important candidate genes that exhibited significant expression in relevant tissues based on in silico analysis, suggesting their potential roles in the respective traits. This study provides a foundation for further research, with the identified candidate genes offering promising targets for molecular breeding programs after validation through marker analysis. Additionally, these genes can be explored through gene cloning to gain deeper insights into the underlying genetic variation and trait regulation.

## Supporting information

S1 Table List of 142 *B. juncea* genotypes used for Genome Wide Association Studies. (DOCX)

S2 Table List of 15219 SNP markers used for Genome Wide Association Studies. (XLSX)

S3 Table Meteorological data recorded during growing period in *rabi* 2020–21 and 2021–22. (XLSX)
