# Supplementary material for: Genome-wide association study uncovers key genomic regions governing agro-morphological and quality traits in Indian mustard [Brassica juncea (L.) Czern. and Coss.]
Source: PLoS One. 2025 Apr 24;20(4):e0322120. doi: 10.1371/journal.pone.0322120 (PMC12021429; doi:10.1371/journal.pone.0322120)
Supplement: S1 Table — (DOCX) [file pone.0322120.s001.docx]

**Genome-wide association study uncovers key genomic regions governing agro-morphological and quality traits in Indian mustard [*Brassica juncea* (L.) Czern. and Coss.]**

Manoj Kumar Patel^1^, Navinder Saini^1*^, Yashpal Taak^1^, Sneha Adhikari^1^, Rajat Chaudhary^1^, Priya Pardeshi^1^, Sudhakar Reddy Basu^1^, Masochon Zimik^1^, Sangita Yadav^2^, K. K. Vinod^1^, Sujata Vasudev^1^, Devendra Kumar Yadava^3*^

^1^Division of Genetics, ICAR-Indian Agricultural Research Institute, New Delhi, India

^2^Division of Seed Science and Technology, ICAR-Indian Agricultural Research Institute, New Delhi, India

^3^Indian Council of Agricultural Research, New Delhi, India

*Corresponding authors

E-mail: [dkygenet@gmail.com](mailto:dkygenet@gmail.com) (DKY)

Email: [navin12@gmail.com](mailto:navin12@gmail.com) (NS)

**SUPPORTING INFORMATION**

**S1 Table: List of 142 *B. juncea* genotypes used for Genome Wide Association Studies**

| **SN** | **Genotypes** | **Remarks** |
| --- | --- | --- |
| 1 | RE-2-2 | East European germplasm |
| 2 | RE-8 | East European germplasm |
| 3 | RE-13 | East European germplasm |
| 4 | RE-35-4 | East European germplasm |
| 5 | CN-101849 | Introduced from Canada Gene Bank |
| 6 | CN-105308 | Introduced from Canada Gene Bank |
| 7 | CN-101846 | Introduced from Canada Gene Bank |
| 8 | CN-112920 | Introduced from Canada Gene Bank |
| 9 | CN-105310 | Introduced from Canada Gene Bank |
| 10 | CN-101834 | Introduced from Canada Gene Bank |
| 11 | CN-105313 | Introduced from Canada Gene Bank |
| 12 | CN-105311 | Introduced from Canada Gene Bank |
| 13 | CN-101887 | Introduced from Canada Gene Bank |
| 14 | DRMRIJ-17-42 | Advanced Breeding Line |
| 15 | DRMRIJ-17-46 | Advanced Breeding Line |
| 16 | DJ-33 | Derived *B. juncea* Line |
| 17 | DJ-57 | Derived *B. juncea* Line |
| 18 | DJ-86 | Derived *B. juncea* Line |
| 19 | DJ-109 | Derived *B. juncea* Line |
| 20 | DTM-4 | Resynthesized determinate mustard |
| 21 | DTM-25 | Resynthesized determinate mustard |
| 22 | DTM-50 | Resynthesized determinate mustard |
| 23 | IM-3 | Introgressed mustard |
| 24 | IM-17 | Introgressed mustard |
| 25 | IM-46 | Introgressed mustard |
| 26 | IM-59 | Introgressed mustard |
| 27 | IM-85 | Introgressed mustard |
| 28 | IM-97 | Introgressed mustard |
| 29 | IM-110 | Introgressed mustard |
| 30 | RE-15 | East European germplasm |
| 31 | AJ-11 | Australian *B. juncea* |
| 32 | RE-7-1 | East European germplasm |
| 33 | RE-11 | East European germplasm |
| 34 | PR-2001-42 | Advanced Breeding line |
| 35 | EM-1 | Advanced Breeding line |
| 36 | IM-108 | Introgressed mustard |
| 37 | PBR-97 | Advanced Breeding line |
| 38 | RE-44 | East European germplasm |
| 39 | PCR-9403 | Advanced Breeding line |
| 40 | KDM-49-1 | Advanced Breeding line |
| 41 | CN-105257 | Introduced from Canada Gene Bank |
| 42 | CN-105312 | Introduced from Canada Gene Bank |
| 43 | CN-105309 | Introduced from Canada Gene Bank |
| 44 | RGN-34 | Advanced Breeding line |
| 45 | NRCQR-9901 | Advanced Breeding line |
| 46 | RGN-73 | Advanced Breeding line |
| 47 | IM-76 | Introgressed mustard |
| 48 | IM-152 | Introgressed mustard |
| 49 | IM-39 | Introgressed mustard |
| 50 | IC-597869 | Indigenous collection |
| 51 | IC-597873 | Indigenous collection |
| 52 | VASUNDHRA | Indian Variety |
| 53 | IM-170 | Introgressed mustard |
| 54 | CN-101813 | Introduced from Canada Gene Bank |
| 55 | CN-101845 | Introduced from Canada Gene Bank |
| 56 | I-79 (M) | Introgression Line |
| 57 | TN-3 | Advanced Breeding line |
| 58 | PM 22 | Indian Variety single zero type |
| 59 | PM 29 | Indian Variety single zero type |
| 60 | LES-42 | Advanced breeding line |
| 61 | PM 30 | Indian Variety single zero type |
| 62 | PM-32 (LES-54) | Indian Variety single zero type |
| 63 | RLC-2 (ELM-123) | Indian variety |
| 64 | RH-801 | Indian variety |
| 65 | ELM-132 | Advanced Breeding line |
| 66 | ALM-936A | Advanced Breeding line |
| 67 | PUSA KARISHMA | Indian variety |
| 68 | PM-24 | Indian variety |
| 69 | TERI | Advanced Breeding line |
| 70 | EC-597318 | Advanced Breeding line |
| 71 | PM-31 (PDZ-1) | Indian Variety double zero type |
| 72 | PDZ-6 | Advanced Breeding Line |
| 73 | PM-33 (PDZ-11) | Indian Variety double zero type |
| 74 | AGRANI | Indian variety |
| 75 | PM-25 | Indian variety |
| 76 | PM-27 | Indian variety |
| 77 | NPJ-245 | Advanced Breeding Line |
| 78 | NPJ-230 | Advanced Breeding Line |
| 79 | PM 26 | Indian variety |
| 80 | NRCHB-101 | Indian variety |
| 81 | PUSA MAHAK | Indian variety |
| 82 | PM-28 | Indian variety |
| 83 | NPJ-161 | Advanced Breeding Line |
| 84 | PUSA JAIKISAN | Indian variety |
| 85 | PUSA TARAK | Indian variety |
| 86 | KRANTI | Indian variety |
| 87 | PM 25M | Mutant selection |
| 88 | JC-33 | Advanced Breeding line |
| 89 | LAXMI | Indian variety |
| 90 | VARUNA | Indian variety |
| 91 | PUSA BAHAR | Indian variety |
| 92 | RB-50 | Indian variety |
| 93 | RH-30 | Indian variety |
| 94 | RH-1499-30 | Advanced Breeding line |
| 95 | RH-1566 | Advanced Breeding line |
| 96 | RH-1723 | Advanced Breeding line |
| 97 | RH-1745 | Advanced Breeding line |
| 98 | RH-761 | Advanced Breeding line |
| 99 | NRCDR-2 | Indian variety |
| 100 | NPJ-181 | Advanced Breeding Line |
| 101 | JC-21 | Advanced Breeding Line |
| 102 | RH-749 | Indian variety |
| 103 | RH 725 | Indian variety |
| 104 | BPR-543-2 | Advanced Breeding Line |
| 105 | BPR-541-4 | Advanced Breeding Line |
| 106 | HEERA | Quality germplasm |
| 107 | BIO YSR | Indian Germplasm |
| 108 | RMM-12-3-18 | Advanced Breeding Line |
| 109 | KMR(E)-19-1 | Advanced Breeding Line |
| 110 | DRMRCI-116 | Advanced Breeding Line |
| 111 | BAUM-09-12-1 | Advanced Breeding Line |
| 112 | PRE-17-5 | Advanced Breeding Line |
| 113 | DONSKAJA | East European mustard |
| 114 | BEC-144 | East European mustard |
| 115 | RH-1999-42 | Advanced Breeding Line |
| 116 | TM-52 | Advanced Breeding Line |
| 117 | KMR[E]-19-2 | Advanced Breeding Line |
| 118 | ORM-41-3-5 | Advanced Breeding Line |
| 119 | RH 1999-18 | Advanced Breeding Line |
| 120 | BAUM-08-14 | Advanced Breeding Line |
| 121 | DRMR-2017-21 | Advanced Breeding Line |
| 122 | PUSA BARANI | Indian variety |
| 123 | NPJ-214 | Advanced Breeding Line |
| 124 | TM-53 | Advanced Breeding Line |
| 125 | NPJ-176 | Advanced Breeding Line |
| 126 | PUSA JAGANNATH | Indian variety |
| 127 | PUSA BOLD | Indian variety |
| 128 | RHUR-2-1 | Advanced Breeding line |
| 129 | RC-371-1 | Indian Germplasm |
| 130 | RC-132 | Indian Germplasm |
| 131 | RJ-10 | Indian Germplasm |
| 132 | IC-597949 | Indigenous collection |
| 133 | RC-571 | Indigenous collection |
| 134 | JM-06010-1 | Australian *B. juncea* |
| 135 | IC-597904 | Indigenous collection |
| 136 | IC-597910 | Indigenous collection |
| 137 | RC-891-1 | Indian Germplasm |
| 138 | GR-325 | German Introduction |
| 139 | IC-597881 | Indigenous collection |
| 140 | RC-1270 | Indian Germplasm |
| 141 | DRMRIJ-31 | Indian Variety |
| 142 | RLC-3 | Indian Variety double zero type |
